# A retrospective cohort study of treatment patterns among patients with metastatic soft tissue sarcoma in the US

**DOI:** 10.1186/s13569-017-0084-4

**Published:** 2017-11-09

**Authors:** Victor M. Villalobos, Stacey DaCosta Byfield, Sameer R. Ghate, Oluwakayode Adejoro

**Affiliations:** 1 University of Colorado Anschutz Medical Campus, 1665 Aurora Ct ACP 5329 Mail Stop F704 Aurora, Denver, CO 80045 USA; 2 Optum, MN101-E300, 11000 Optum Circle, Eden Prairie, MN 55344 USA; 3 Novartis, One Health Plaza 345/5130B, East Hanover, NJ 07936 USA

**Keywords:** Metastatic soft tissue sarcoma, Treatment patterns, Pazopanib

## Abstract

**Background:**

Since treatment patterns in metastatic soft tissue sarcoma (mSTS) have not been studied subsequent to US approval of pazopanib in 2012, this study sought to examine mSTS treatment patterns by line of therapy, including regimen and duration of therapy.

**Methods:**

This retrospective study employed administrative claims from a large US health plan from 1/2006–9/2015. Adult mSTS patients were required to have an NCCN-recommended therapy and be continuously enrolled in the health plan during the study period. The most frequent regimens for distinct lines of therapy (LOT) were assessed. Sensitivity analyses evaluated changes to study findings using two alternate medical and pharmacy claims diagnostic algorithms to define the STS study population.

**Results:**

Among 555 patients with mSTS, mean age was 59 years and 54% were male. During the study period, 41% of patients initiated ≥ 2 LOTs; 16% had ≥ 3 LOTs and 5% had ≥ 4 LOTs. Docetaxel + gemcitabine was most common in LOT1, pazopanib in LOT2 and LOT3, and doxorubicin in LOT4. The five most common LOT1 regimens represented 53% of patients; among the remaining 47%, the most common regimen represented < 6% of patients. Among patients with pazopanib in LOT2 and LOT3, the most common prior regimen was docetaxel + gemcitabine (47% and 30% respectively). Kaplan–Meier estimation of median treatment duration overall for LOT1 was 3.5 months, while for LOT2 and LOT3, median treatment duration was 2.9 and 3.3 months, respectively. For both sensitivity analyses, patient demographic and clinical characteristics were similar to the original study population, and the five most frequently used regimens in LOT1 and LOT2 were similar among the three populations regardless of the population selection criteria employed.

**Conclusion:**

Choice of regimen by LOT among patients with mSTS is varied; < 65% of patients in any LOT received the five most common regimens. Pazopanib, the only approved targeted therapy, is primarily used in second and later lines of therapy and is mostly given post docetaxel + gemcitabine.

## Background

Soft tissue sarcoma (STS) is a heterogeneous group of uncommon tumors that arise from mesenchymal cells at connective tissue body sites which differ by various inherent features including histology, molecular genetic profiles, site predilection, and outcomes of care [1]. STS includes approximately 40 malignant histological subtypes, and is most prominent in the extremities (50%), trunk and retroperitoneum (40%), and head and neck (10%). STS represent ~ 1% of all adult malignancies [2]. An estimated 11,930 new cases of STS were diagnosed in 2015 in the US [3]. STS is associated with high mortality; an estimated 4870 deaths due to STS were estimated to occur in the US in 2015 [3].

The choice of chemotherapy in metastatic STS (mSTS) should be individualized based on empirical knowledge of the chemosensitivity of various histologic subtypes and biology of the tumor [4–6]. Most sarcomas are sensitive to gemcitabine/docetaxel and doxorubicin; angiosarcomas are sensitive to taxanes, liposarcomas are sensitive to doxorubicin-based regimens, synovial sarcomas are sensitive to ifosfamide and leiomyosarcomas are sensitive to gemcitabine, doxorubicin, ifosfamide, and trabectedin [4, 7, 8]. Pazopanib, the first targeted therapy for STS, was approved by the US Food and Drug Administration (FDA) in April 2012 for mSTS among patients with prior chemotherapy [9]; subsequently, olaratumab was approved in the US in October 2016 for treatment of soft tissue sarcomas [10]. National Comprehensive Cancer Network (NCCN) treatment recommendations include single agents (including dacarbazine, doxorubicin and ifosfamide), anthracycline-based combination regimens, or targeted therapy with pazopanib for relapsed or advanced/metastatic disease [2]. Choice of chemotherapy is influenced by both the stage of disease and practitioner preference. Single agent therapies are typically used in the metastatic setting, whereas chemotherapy combinations are generally used in the neoadjuvant/adjuvant setting or in settings where response is favored.

Several studies evaluating treatment patterns in STS have been published which precede pazopanib’s availability in the US. The retrospective Sarcoma Treatment and Burden of Illness in North America and Europe (SABINE) study of treatment patterns in North America and Europe found that doxorubicin monotherapy or an anthracycline plus ifosfamide were the most common first-line treatments in metastatic/relapsed STS, and the most common second-line treatment was gemcitabine plus docetaxel [11]. A second US-based study found doxorubicin plus ifosfamide or docetaxel plus gemcitabine accounted for a combined 53% of first-line treatment in mSTS, and docetaxel plus gemcitabine accounted for 52% of second-line treatment [12]. Finally, a study conducted in the US of patients with metastatic/relapsed STS found that 44% received anthracycline-based and 28% received gemcitabine-based first-line regimens; gemcitabine-based (28%) and anthracycline-based (24%) regimens were most commonly used second-line, and no consistent regimens were used beyond second line of therapy (LOT) [13].

Large medical and pharmacy claims databases, which are used for billing and payment purposes, are useful to examine “real-world” practice patterns across different treatment settings within the US. Due to the large numbers of patients covered within these databases, they are particularly valuable to evaluate treatment for relatively rare conditions. Since prior published evaluations of treatment patterns in mSTS preceded pazopanib’s US approval date, current treatment patterns among patients with mSTS are unknown. The primary objective of our study was to examine treatment patterns by  LOT, including regimen and duration of therapy, in a large US administrative claims database. The secondary objective of this study was to identify and describe characteristics of patients who initiated therapy for mSTS.

## Methods

This was a retrospective cohort study which employed medical and pharmacy claims from the Optum Research Database (ORD). The ORD contains inpatient, outpatient, and pharmacy claims for commercial enrollees with both medical and pharmacy benefit coverage from a health plan affiliated with Optum from 1993 to the present. For 2013, data relating to approximately 12.7 million individuals (~ 4% of the US population in 2013) with both medical and pharmacy benefit coverage are available. In addition, ORD also contains inpatient, outpatient, and pharmacy claims for approximately 4.2 million enrollees in Medicare Part C (commonly referred as Medicare Advantage program) since 2006. Social Security Administration (SSA) Death Master Files were also used to supplement patient death information, if applicable.

### Cohort selection criteria

The study included data from January 1, 2006 through September 30, 2015. Eligible patients had commercial or Medicare Advantage insurance with both medical and pharmacy benefits. Patients were required to have had at least one prescription for NCCN-recommended systemic anticancer therapy (Appendix 1: Table 3) for mSTS management during the study identification period (May 1, 2012 through August 31, 2015). The index date was the start date of the first LOT  for treatment of mSTS during the identification period. All available patient baseline data from January 1, 2006 through the index date was extracted to ascertain initial treatment and evidence of metastatic disease. Patients were required to have had at least two non-diagnostic medical claims in any position for STS [non-gastrointestinal stromal tumors (GIST)] [International Classification of Diseases, 9th revision, Clinical Modification (ICD-9-CM) 171.xx], at least 30 days apart, during the identification period. The study population was limited to patients 18 years of age or older as of the index date. Patients were required to be newly treated for metastasis, defined as follows: (1) at least one claim with a diagnosis code for metastasis (ICD-9-CM 196.x, 197.x, 198.x, 199.0) in any position during the variable baseline through follow-up period; the date of the first claim with a metastasis diagnosis was termed the ‘met date’, (2) the ‘met date’ was required to be prior to or on the index date (the start of the first LOT during the identification period) with no claims for systemic anticancer therapy between the ‘met date’ and the index date, (3) no LOTs with claims for metastatic disease prior to the index date, (4) patients were continuously benefit-eligible from at least 6 months preceding the ‘met date’, through the index date and for one or more months after the index date, and (5) at least one claim for STS was required prior to or on the ‘met date’. A cohort identification timeline is included in Appendix 2: Fig. 4. To exclude GIST, patients with one or more claims for imatinib during the study identification period were eliminated from the final study population. Patient treatment cohorts were determined based on the most common regimens received during the first LOT.

### Independent study variables

Baseline patient demographic and clinical characteristics were evaluated for the study population based on health plan administrative claims and enrollment data. Demographic characteristics such as age as of index year, gender, and geographic region of health plan were assessed. Baseline clinical characteristics included Quan-Charlson comorbidity score [14], a composite comorbidity score (higher score = greater comorbidity burden) calculated based on assignment of a point value for specific diagnosis codes on medical claims during the pre-index period corresponding to chronic disease states. For example, a ‘healthy’ patient with no comorbid conditions would have a total composite score of 0.0. Metastatic disease is associated with a point value of 6.0; a patient with claims history consistent with metastatic disease but no other comorbid conditions would have a composite score of 6.0. Radiation during the baseline period was evaluated using procedure codes [Current Procedural Terminology (CPT), Healthcare Common Procedure Coding System (HCPCS), Medicare diagnosis-related group (MS-DRG), revenue, and ICD-9-CM procedure codes]. History of surgical procedure(s) during baseline was captured using CPT, HCPCS, and ICD-9-CM procedure codes.

### Study endpoints

Primary outcomes included specific anticancer medication regimens by LOT and duration of therapy. Up to four LOT regimens were assessed. A specific LOT began on the date of the first infusion or fill for a systemic anticancer agent, and the regimen associated with this LOT included all anti-cancer agents received within 45 days following the first infusion or fill date. This specific LOT continued until the earliest of any of the following: (1) addition or substitution of a new agent after the initial agent(s) (LOT end date defined as the day prior to the start of the new agent, but discontinuation of one agent from a multidrug regimen did not qualify as ending LOT); (2) a treatment gap ≥ 60 days after the runout date of all agents in the LOT [LOT end date was the runout date prior to the gap; runout date was defined for infused/injected drugs as the latest date of administration + 29 days, and for drugs obtained through pharmacy benefit runout date was fill date + (days supply − 1)]; (3) death; or (4) disenrollment or end of the study period. A LOT was considered censored if the LOT ended due to end of study period or disenrollment. Second through fourth LOTs were identified by the initiation of anti-cancer therapy after the end of the previous LOT. Note that a re-initiation of a previous regimen would be considered a new LOT as long as criterion #2 above (a treatment gap of ≥ 60 days) was met. The algorithm described above applied to the other LOTs and the number of LOTs examined was dependent on available sample size. The most common regimens by LOT were identified, and a count of LOTs was computed.

### Statistical methods

Descriptive statistics of all variables were calculated for the mSTS population, including means and 95% confidence intervals (CI) for continuous variables, and frequency tables and percentages for categorical variables. Global comparisons of patient characteristics were made across LOT1 regimens and included Chi square testing for frequency tables and analysis of variance for continuous variables. Duration of therapy for each LOT was summarized with arithmetic means and using Kaplan–Meier methods.

### Sensitivity analyses

To investigate the potential impact of the inadvertent inclusion of patients without mSTS in our study population due to limitations associated with using medical claims to identify patients with mSTS, two sensitivity analyses evaluated changes to study findings when more restrictive claims diagnostic criteria were applied to STS population identification. The first sensitivity analysis excluded patients who received atypical anticancer agents (bevacizumab, carboplatin, paclitaxel) during the study period. The second sensitivity analysis excluded both patients who used atypical anticancer agents (bevacizumab, carboplatin, paclitaxel) and patients with one or more claims with a non-STS cancer diagnosis in the first or second position in conjunction with a code for an injectable systemic anticancer therapy on the same claim. Descriptive analyses were performed on both study subpopulations.

## Results

**Fig. 1:**
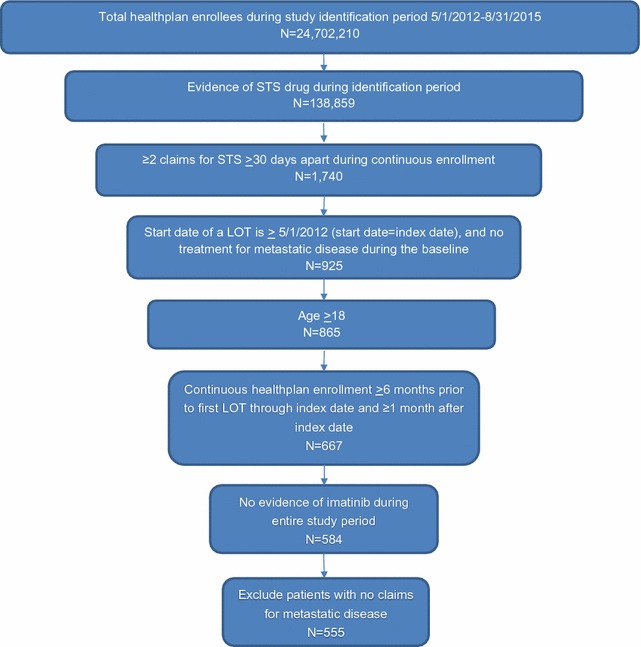
Study sample attrition diagram

A total of 138,859 patients were prescribed an STS drug during the study identification period, and 1740 (1.3%) had at least two claims for STS at least 30 days apart during continuous enrollment (Fig. 1). Subsequent to application of all other cohort inclusion and exclusion criteria, 555 patients with mSTS were identified for the final study cohort. Mean age overall was 58.8 (SD 16.3) years and 46.3% were female (Table 1). Among the mSTS study cohort, 41.3% had 2 LOTs, 15.9% had 3 LOTs, and 5.0% had 4 LOTs during the study period. Across all LOTs, the most frequently prescribed agent (in either mono- or combination therapy) was gemcitabine (43%), followed by doxorubicin (36%), docetaxel (35%), and pazopanib (21%) (Appendix 3: Fig. 5).

**Table 1:** Patient baseline demographic and clinical characteristics

	Total	First LOT						
		GEM + DOC	DOX	PAZO	DOX + IFO	GEM	Others	Overall p value
N (%)	555 (100)	124 (22.3)	72 (13.0)	38 (6.8)	31 (5.6)	29 (5.2)	261 (47.0)	
Age, mean (95% CI)	58.8 (57.5–60.2)	57.1 (54.9–59.3)	65.9 (62.5–69.3)	59.0 (53.3–64.7)	54.7 (48.9–60.4)	66.3 (61.5–71.1)	57.4 (55.2–59.5)	< 0.001
Age categories, n (%)								
18–44	108 (19.5)	17 (13.7)	6 (8.3)	11 (29.0)	10 (32.3)	1 (3.5)	63 (24.1)	< 0.001
45–54	91 (16.4)	30 (24.2)	9 (12.5)	3 (7.9)	3 (9.7)	4 (13.8)	42 (16.1)	0.091
55–64	132 (23.8)	42 (33.9)	16 (22.2)	8 (21.1)	7 (22.6)	6 (20.7)	53 (20.3)	0.104
65–74	128 (23.1)	28 (22.6)	19 (26.4)	8 (21.1)	10 (32.3)	8 (27.6)	55 (21.1)	0.708
75+	96 (17.3)	7 (5.7)	22 (30.6)	8 (21.1)	1 (3.2)	10 (34.5)	48 (18.4)	< 0.001
Female, n (%)	257 (46.3)	75 (60.5)	36 (50.0)	21 (55.3)	10 (32.3)	13 (44.8)	102 (39.1)	0.001
Coverage type, n (%)								
Commercial	363 (65.4)	90 (72.6)	38 (52.8)	28 (73.7)	22 (71.0)	15 (51.7)	170 (65.1)	0.036
Aged < 65 years	318 (87.6)	85 (94.4)	30 (79.0)	22 (78.6)	20 (90.9)	10 (66.7)	151 (88.8)	0.010
Aged 65+ years	45 (12.4)	5 (5.6)	8 (21.1)	6 (21.4)	2 (9.1)	5 (33.3)	19 (11.1)	0.010
Medicare advantage	192 (34.6)	34 (27.4)	34 (47.2)	10 (26.3)	9 (29.0)	14 (48.3)	91 (34.9)	0.036
Geographic region, n (%)								
Northeast	92 (16.6)	17 (13.7)	11 (15.3)	7 (18.4)	8 (25.8)	6 (20.7)	43 (16.5)	0.672
Midwest	151 (27.2)	32 (25.8)	24 (33.3)	10 (26.3)	8 (25.8)	9 (31.0)	68 (26.1)	0.860
South	228 (41.1)	57 (46.0)	25 (34.7)	17 (44.7)	9 (29.0)	9 (31.0)	111 (42.5)	0.313
West	84 (15.1)	18 (14.5)	12 (16.7)	4 (10.5)	6 (19.4)	5 (17.2)	39 (14.9)	0.931
Quan-Charlson comorbidity score, mean (95% CI)	7.6 (7.4–7.7)	7.4 (7.1–7.7)	7.7 (7.4–8.1)	7.6 (6.9–8.3)	6.9 (6.5–7.4)	8.0 (7.3–8.6)	7.6 (7.4–7.8)	0.166
≥ 1 Radiation claim, n (%)	251 (45.2)	37 (29.8)	44 (61.1)	18 (47.4)	9 (29.0)	14 (48.3)	129 (49.4)	< 0.001
≥ 1 Surgery claim, n (%)	397 (71.5)	96 (77.4)	53 (73.6)	28 (73.7)	20 (64.5)	20 (69.0)	180 (69.0)	0.544
Length of follow-up (days), mean (95% CI)	325.8 (303.6–347.9)	363.6 (313.9–413.3)	316.0 (261.8–370.2)	317.8 (234.6–401.0)	365.4 (254.9–475.8)	186.1 (121.3–251.0)	322.4 (289.6–355.3)	0.043

*DOC* docetaxel, *DOX* doxorubicin, *GEM* gemcitabine, *IFO* ifosfamide, *PAZO* pazopanib, *SD* standard deviation

**Fig. 2:**
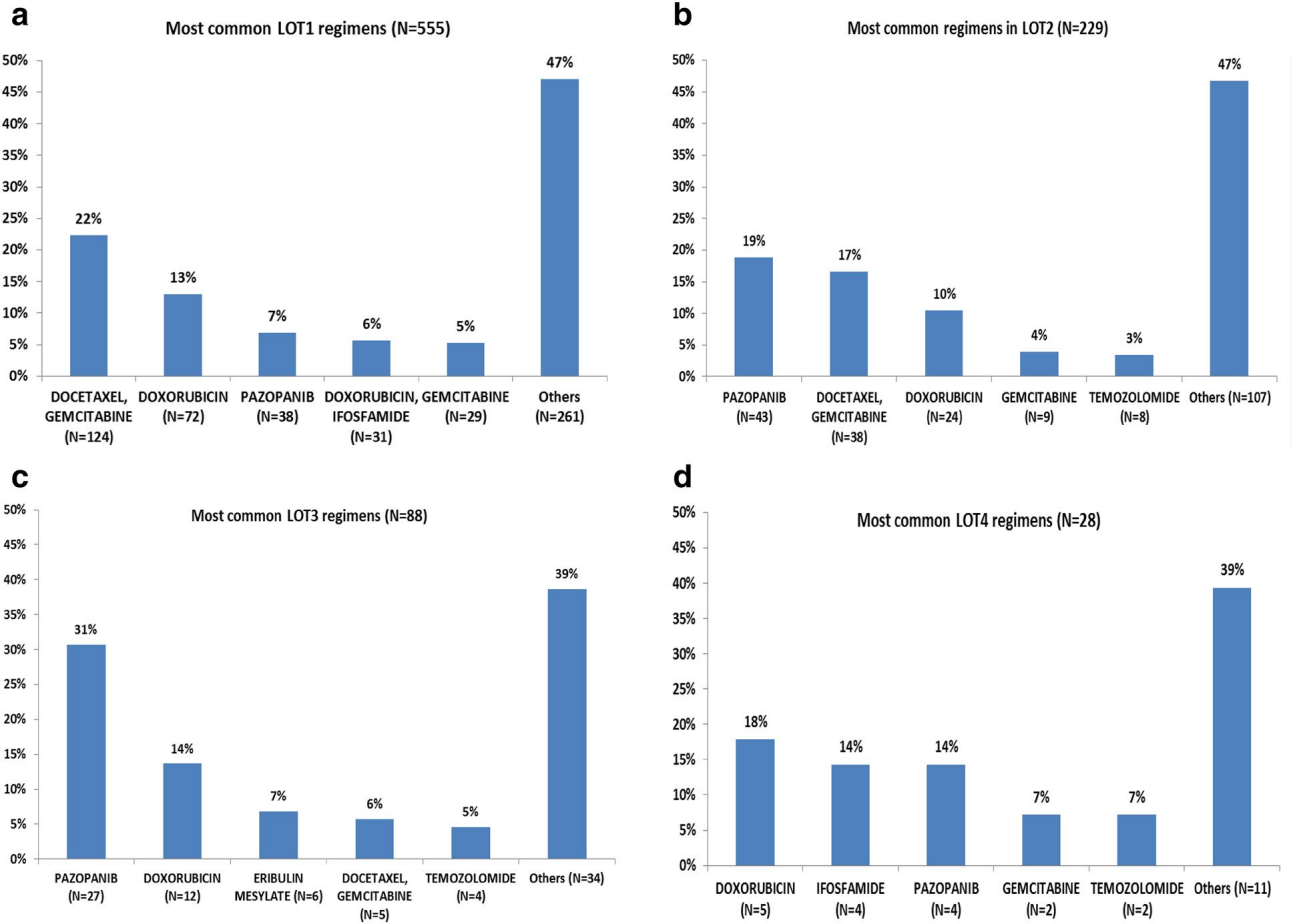
Most common therapeutic regimens by LOT among patients with mSTS. **a** Most common regimens for first LOT (N = 555). **b** Most common regimens for second LOT (N = 229). **c** Most common regimens for third LOT (N = 88). **d** Most common regimens for fourth LOT (N = 28)

The most frequent LOT1 regimen was docetaxel + gemcitabine (22.3%), followed by doxorubicin (13.0%) (Fig. 2a). There was considerable variation in LOT1 regimens, as almost half (47%) of patients had ‘other’ first-line therapies, and among these patients, each specific regimen accounted for < 6% of all patients (Appendix 4: Table 4). There were significant differences in mean age and gender by LOT1 regimen (Table 1). About 2 in 3 patients (65.4%) were covered by commercial insurance, while 34.6% were covered by Medicare Advantage. Geographic distribution of the mSTS cohort was consistent with the overall distribution of all health plan enrollees. Mean Quan-Charlson comorbidity score was 7.6 for the mSTS cohort and was similar regardless of LOT1 regimen. Almost half (45.2%) of patients overall had radiation during the variable baseline period, and 71.5% had evidence of a surgical procedure. Overall mean duration of follow-up was 325.8 days (SD 265.3).

The most common therapeutic regimens by LOT are shown in Fig. 2. Fewer than 65% of patients in any LOT received one of the top 5 most common regimens. In LOT2 (Fig. 2b), pazopanib (19%) was the most frequent regimen, followed by docetaxel + gemcitabine (17%). Pazopanib (31%) was also the most common LOT3 regimen, followed by doxorubicin (14%), while doxorubicin (18%) was most common in LOT4, followed by ifosfamide (14%) and pazopanib (14%) (Fig. 2c). A list of medications included in the ‘other’ category for LOT1–LOT3 is included in Appendix 4: Table 4.

**Fig. 3:**
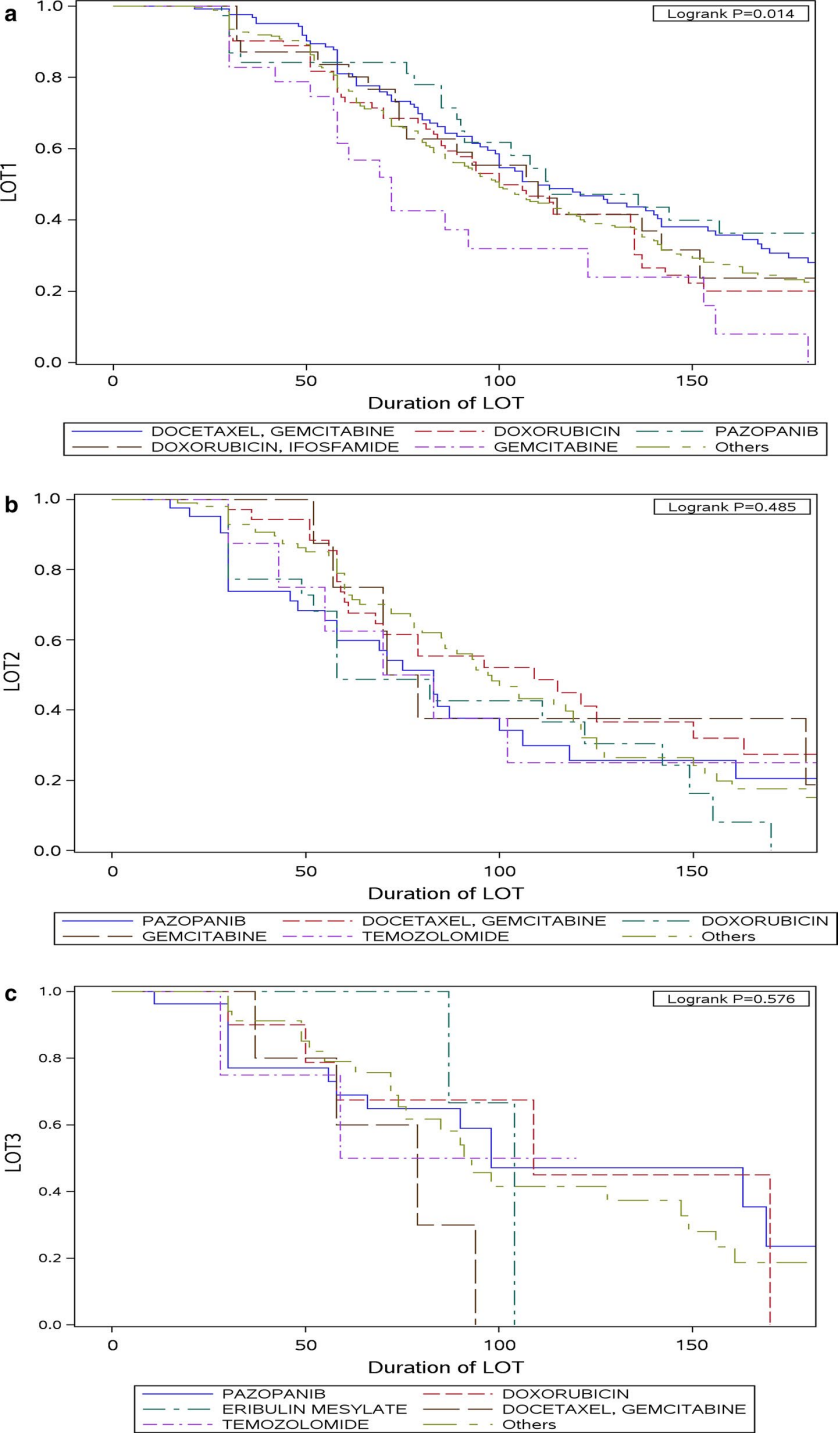
Duration of first, second and third LOTs. **a** Duration of therapy for LOT1 by treatment regimen. **b** Duration of therapy for LOT2 by treatment regimen. **c** Duration of therapy for LOT3 by treatment regimen

**Fig. 4:**
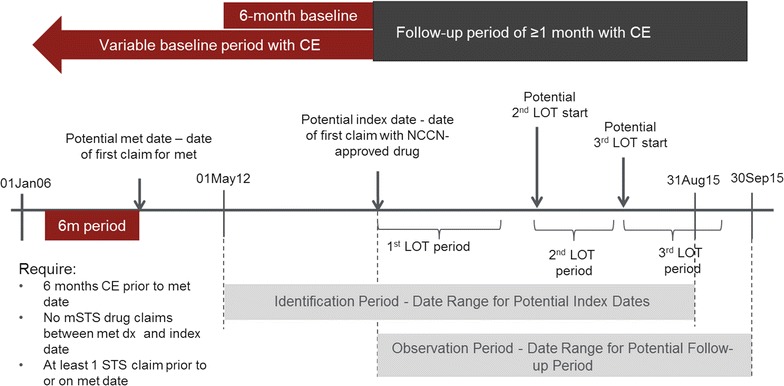
Study timeline and identification of patients with metastatic STS

**Fig. 5:**
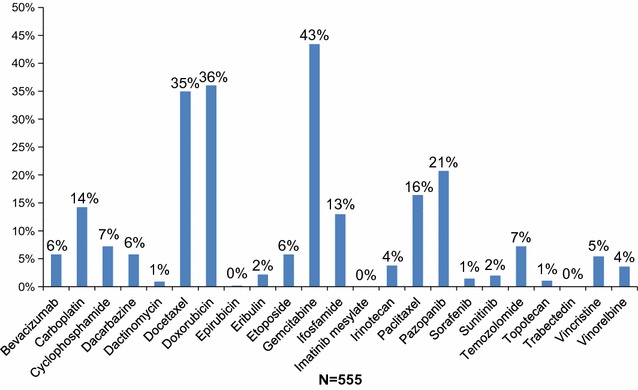
Most  common  NCCN-recommended agents received during follow-up

Mean (median) treatment duration overall for LOT1 was 3.7 months (2.9 months), while for LOT2 and LOT3, mean treatment duration was 2.8 (2.3) and 3.0 (2.6) months, respectively. Mean (median) duration of therapy for patients initially treated with pazopanib was 5.2 (3.1) months. Figure 3 depicts Kaplan–Meier estimates for LOT1, LOT2, and LOT3; Kaplan–Meier estimation of median duration of therapy for LOT1, LOT2 and LOT3 were 3.5, 2.9 and 3.3 months, respectively. There was a significant difference in the duration of LOT1 by regimen (global p value = 0.014) (Fig. 3a) while for LOT2 and LOT3, median treatment durations by respective LOT regimens were similar (Fig. 3b, c).

Among patients with more than one LOT during the study period (n = 229), the most frequently prescribed LOT2 regimen was pazopanib (n = 43; 19% of 229 patients) (Table 2). Most patients treated with pazopanib in LOT2 used docetaxel + gemcitabine (n = 20; 47% of 43 patients) in LOT1. Among patients with more than 2 LOTs during the study period (n = 88), of the patients who received pazopanib as their LOT3 regimen (n = 27; 31% of 88 patients), 30% used docetaxel + gemcitabine for LOT2 (n = 8), and 15% each (n = 4) used pazopanib or doxorubicin. A small number of patients received the same medication regimen for sequential LOTs; for example, five patients were treated with docetaxel + gemcitabine for both LOT1 and LOT2 (Table 2).

**Table 2:** LOT transition among patients with > 1 LOT

LOT1	LOT2 (N = 229)											
	Pazopanib		Docetaxel, gemcitabine		Doxorubicin		Gemcitabine		Temozolomide		Others	
	(N = 43, 18.8%)		(N = 38, 16.6%)		(N = 24, 10.5%)		(N = 9, 3.9%)		(N = 8, 3.5%)		(N = 107, 46.7%)	
	n	%	n	%	n	%	n	%	n	%	n	%
Docetaxel, gemcitabine	20	46.5	5	13.2	14	58.3	0	0.0	0	0.0	19	17.8
Doxorubicin	4	9.3	12	31.6	1	4.2	1	11.1	2	25.0	13	12.2
Pazopanib	3	7.0	4	10.5	1	4.2	3	33.3	2	25.0	4	3.7
Doxorubicin, ifosfamide	3	7.0	5	13.2	0	0.0	1	11.1	0	0.0	2	1.9
Gemcitabine	2	4.7	0	0.0	0	0.0	0	0.0	0	0.0	6	5.6
Others	11	25.6	12	31.6	8	33.3	4	44.4	4	50.0	63	58.9

LOT2	LOT3 (N = 88)											
	Pazopanib		Doxorubicin		Eribulin mesylate		Docetaxel, gemcitabine		Temozolomide		Others	
	(N = 27, 30.7%)		(N = 12, 13.6%)		(N = 6, 6.8%)		(N = 5, 5.7%)		(N = 4, 4.5%)		(N = 34, 38.6%)	
	n	%	n	%	n	%	n	%	n	%	n	%
Pazopanib	4	14.8	5	41.7	0	0.0	0	0.0	2	50.0	5	14.7
Docetaxel, gemcitabine	8	29.6	2	16.7	0	0.0	1	20.0	0	0.0	2	5.9
Doxorubicin	4	14.8	1	8.3	1	16.7	1	20.0	0	0.0	4	11.8
Gemcitabine	1	3.7	2	16.7	0	0.0	0	0.0	0	0.0	2	5.9
Temozolomide	2	7.4	0	0.0	0	0.0	1	20.0	0	0.0	3	8.8
Others	8	29.6	2	16.7	5	83.3	2	40.0	2	50.0	18	52.9

### Sensitivity analyses

Patient demographic and clinical characteristics were similar across the original population and both sensitivity I and sensitivity II populations (data not shown); minor differences can be explained by new exclusionary criteria used to restrict the study population. The five most frequently used regimens in LOT1 and LOT2 were similar among the three populations regardless of the population selection criteria. Across all three study populations, the lowest percentage of patients (32%) were classified in the ‘other’ medications category in the sensitivity I population.

## Discussion

We found wide variation in the regimens used across all LOTs for the treatment of mSTS. In LOT1, only 53% of patients combined were treated with the 5 most common LOT1 regimens, and about 47% of patients received a regimen other than these regimens. The most commonly used regimen within the ‘other’ category accounted for < 6% of the STS population. Our findings may, in part, be explained by the numerous histological types (> 40) of STS, varying chemosensitivity of histological types, disease progression, and the lack of evidence on the optimal treatment and sequence of treatment of each histology. It is also possible that our results reflect variable clinician expertise with and adherence to recommended chemotherapy treatment guidelines [2]. Consistent with the findings of Wagner et al. [13], no consistent regimens were used beyond second-line treatment in the current study. Docetaxel + gemcitabine, followed by doxorubicin and pazopanib, were the top three regimens in LOT1, and docetaxel + gemcitabine was the first line regimen in 47% of patients who received pazopanib as LOT2 and 58% of patients who received doxorubicin as LOT2. Doxorubicin has generally been the mainstay of first line therapy for most mSTS, due to its lower toxicity and relative ease of administration [15, 16]. The recent GeDDiS trial compared first line treatment of doxorubicin to docetaxel + gemcitabine among patients with advanced or metastatic STS in the United Kingdom and Switzerland. Study investigators concluded that doxorubicin is appropriate as the standard first line treatment for most patients with advanced STS, as compared to docetaxel + gemcitabine, it is less difficult to administer, perceived by patients as less toxic, and less expensive, despite comparable survival outcomes [15]. The addition of olaratumab to first line therapy is likely to solidify the use of doxorubicin in the first line [17]. Prior studies of first-line treatment have found that the most common regimens were doxorubicin monotherapy (34%) or an anthracycline + ifosfamide (30%) [11], anthracycline-based (44%) or gemcitabine-based (28%) [13], doxorubicin (± ifosfamide) (46%) [12], or doxorubicin (± ifosfamide) (66%) [18]. Docetaxel + gemcitabine may be preferred for certain mSTS including leiomyosarcomas and undifferentiated pleomorphic sarcomas (UPS) [16]. Since histology was not captured in the claims data used for the current study, we have no knowledge of whether our study’s results were driven by histologic subtype, and it is unclear whether inconsistent findings between studies, even for preferred first-line regimens, is more related to histologic subtypes represented by the individual study populations or true differences in practice patterns. Doxorubicin alone was more popular than doxorubicin + ifosfamide while docetaxel + gemcitabine were more popular than gemcitabine alone. Published evidence suggests that the therapeutic effect of doxorubicin + ifosfamide is additive with no statistically significant overall survival benefit and is associated with more adverse effects compared to the synergistic relationship between gemcitabine and docetaxel [16, 19, 20].

In the current study, pazopanib, followed by docetaxel + gemcitabine, were the leading regimens in LOT2. Compared to other regimens used in mSTS, pazopanib may be preferred by patients due to its oral route of administration. Clinical trials of pazopanib have found lower rates of certain adverse events (anemia, neutropenia, nausea/vomiting, and elevated AST/ALT) than reported for clinical trials of trabectedin, though anorexia was more common with pazopanib [7, 21, 22]. However, the lack of head-to-head clinical trials comparing these agents and the use of different patient populations to evaluate event rates makes direct comparisons problematic. Trabectedin was not available in the US until late 2015, though it has been commonly used as a second-line treatment in Europe since 2007. Prior to pazopanib’s availability in the US, Wagner et al. (2000–2011) found that gemcitabine-based (28%) and anthracycline-based (24%) regimens were used most often in LOT2 [13]. The most common second-line treatment was docetaxel + gemcitabine (18%) in the SABINE study by Leahy et al. [11]. Also, Chen et al. found that docetaxel + gemcitabine (52%) was the most common regimen in LOT2 [12]. These results suggest widespread use of gemcitabine in LOT2 prior to pazopanib’s US availability, either alone or in combination with docetaxel, and less variation in LOT2 regimen preference across different study populations. In contrast, Bae and colleagues found that 53% used ifosfamide in LOT2 in an Australian advanced STS population, although pazopanib was sporadically used as subsequent LOTin Bae’s study population following its availability in Australia in March of 2014 [18].

To our knowledge, the current study is the first published study of treatment patterns in mSTS since pazopanib became available in the US in 2012. A somewhat unexpected finding of our study was that ~ 7% of patients received pazopanib as initial therapy, as pazopanib is approved as second or later line of management for mSTS in the US. This may represent off-label use of pazopanib, possibly related to early clinical trial results suggesting a role for pazopanib in first-line treatment of soft tissue sarcomas, including solitary fibrous tumor (SFT) and clear cell sarcoma [23–25]. However, it may be that physicians may be more comfortable with this drug early on and consider it to be a more tolerable treatment than other first-line options, such as high dose doxorubicin. Additionally, it is possible that the use of pazopanib as first LOT in our study may be a misclassification of LOT2 as LOT1 regimens using administrative claims data to ascertain LOT. Additionally, another unexpected finding of our analysis was significant usage of carboplatin in our mSTS cohort, since this drug has very limited efficacy in sarcoma. These observations may reflect the somewhat fragmented oncology care for sarcoma within the American health system. While in Europe, most sarcoma patients are referred as standard practice to high-volume referral centers, many patients in the United States are treated in private, non-academic practices. Lack of experience with sarcomas may result in therapeutic choices that do not align with evidence-based best practices and therefore receiving care under the guidance of a high-volume referral center may be important for patients’ receipt of optimal recommendations for therapy.

Treatment with new drugs (different agents from LOT1) was the common strategy during disease progression/subsequent LOTs. Drug rechallenge, the repeat administration of the same regimen which may occur following drug holiday, disease progression or relapse, was low in general, but higher among docetaxel + gemcitabine-treated patients than pazopanib-treated patients, and in later LOTs than earlier LOTs. This may reflect fewer regimen options for subsequent therapy to choose from after selection of initial treatment regimen. Shorter duration of therapy for later LOTs relative to LOT1 as observed in our study could be multifactorial and be suggestive of worsening disease or resistant disease. Longer duration of therapy on pazopanib may suggest ease of use, relative effectiveness, and/or tolerability of pazopanib. Finally, results of sensitivity analyses in more rigorously defined mSTS patient subsets confirmed our overall results.

### Limitations

Our study’s results should be interpreted in the context of several important study limitations. This study relied on identification codes on administrative claims data to determine STS, disease metastasis and the earliest date of pharmacy claims for identifying the initial patient population. The reporting of metastatic disease through the listing of its ICD code on claims by the treating physician may not be done consistently, and it was not possible for us to independently verify via pathological diagnosis; hence there is possibility for misclassification of patients with mSTS. Administrative claims data also do not contain clinical prognostic information (e.g. histologic subtype, clinical stage and extent of disease) of patients. The codification of sarcomas is particularly problematic, since most of the diagnostic subtypes utilize the same ICD-9-CM code. Conducting sub-analyses on patients with prevalent histologic subtypes would have provided better information applicable to patients with similar histologic subtype, but this was not possible using medical claims data. A regimen change may not always be due to disease progression but also other events like adverse events or drug toxicities, which may not be ascertainable using claims data. We used information from the public view of the SSA death files for cohort selection, and these files do not comprehensively capture all deaths. Finally, a medical-claims based algorithm was developed to classify LOT1-4, and may not accurately identify specific LOTs. The LOT algorithm reflects gaps in therapy but, due to the nature of claims data, the reason for these gaps is unknown, and therapy lapses due to tolerability issues, medication persistence and adherence, drug rechallenge, and other clinically relevant therapy gaps may have been classified as distinct LOTs; this may explain why a small number of patients were treated with the same regimens for two sequential LOTs. Furthermore, our LOT algorithm considered all anti-cancer agents administered within 45 days following the first infusion as the initial regimen received, and it is possible that a true subsequent LOT may start within 45 days and represent a distinct LOT but not correctly be classified as such.

## Conclusions

While our study lacked clinical diagnostic information to enable investigation of treatment patterns by histologic subtypes, this is the first study of treatment patterns in mSTS to be conducted since the US approval of pazopanib in 2012, and our findings provide direction for future research in mSTS treatment patterns. We found wide variations in the regimens used in all LOTs for the treatment of mSTS. Fewer than 65% of patients in any LOT received the five most common regimens. In LOT1, approximately 47% of patients received ‘other’ chemotherapy, and the most commonly used regimen within the ‘other’ category accounted for less than 5% of the STS population. Docetaxel + gemcitabine, followed by doxorubicin and pazopanib, were the top three regimens in LOT1, while pazopanib, followed by docetaxel + gemcitabine, were the leading regimens in LOT2. Treatment with new drugs (different agents from LOT1) was the common strategy during disease progression. Pazopanib, the only approved targeted therapy for mSTS, is frequently administered second-line post docetaxel + gemcitabine, was used by 7% of patients in LOT1, and is being adopted in second and later LOTs since its availability in 2012.

## Appendix 1

See Table 3.

**Table 3:** List of NCCN-recommended systemic anticancer therapies

NCCN-recommended systemic cancer therapy
Bevacizumab
Carboplatin
Cyclophosphamide
Dacarbazine
Dactinomycin
Docetaxel
Doxorubicin/pegylated liposomal doxorubicin
Epirubicin
Eribulin
Etoposide
Gemcitabine
Ifosfamide
Irinotecan
Paclitaxel
Pazopanib
Sorafenib
Sunitinib
Temozolomide
Topotecan
Trabectedin
Vincristine
Vinorelbine

## Appendix 2

See Fig. 4.

## Appendix 3

See Fig. 5.

## Appendix 4

See Table 4.

**Table 4:** Frequency of use of specific medications included in ‘Other’ category for LOT1-LOT3

	Frequency	Percent (%)
LOT 1 regimen		
Carboplatin, paclitaxel	29	5.2
Paclitaxel	28	5.0
Carboplatin	18	3.2
Temozolomide	14	2.5
Bevacizumab	12	2.2
Cyclophosphamide, doxorubicin, vincristine	9	1.6
Sunitinib	8	1.4
Docetaxel	8	1.4
Etoposide	7	1.3
Ifosfamide	7	1.3
Etoposide, ifosfamide	6	1.1
Carboplatin, docetaxel	6	1.1
Irinotecan, temozolomide	5	0.9
Sorafenib	5	0.9
Gemcitabine, paclitaxel	4	0.7
Carboplatin, etoposide	4	0.7
Cyclophosphamide	4	0.7
Dacarbazine	4	0.7
Docetaxel, doxorubicin, gemcitabine	4	0.7
Bevacizumab, temozolomide	3	0.5
Bevacizumab, carboplatin, paclitaxel	3	0.5
Vincristine	3	0.5
Vinorelbine	3	0.5
Carboplatin, gemcitabine	3	0.5
Cyclophosphamide, dactinomycin, vincristine	3	0.5
Cyclophosphamide, doxorubicin, etoposide, ifosfamide, vincristine	3	0.5
Dacarbazine, doxorubicin	3	0.5
Eribulin	3	0.5
Gemcitabine, vinorelbine	2	0.4
Irinotecan	2	0.4
Irinotecan, temozolomide, vincristine	2	0.4
Carboplatin, irinotecan	2	0.4
Cyclophosphamide, etoposide	2	0.4
Cyclophosphamide, vinorelbine	2	0.4
Cyclophosphamide, dacarbazine, doxorubicin	2	0.4
Cyclophosphamide, doxorubicin, irinotecan, vincristine	2	0.4
Docetaxel, gemcitabine, pazopanib	2	0.4
Doxorubicin, gemcitabine	2	0.4
Doxorubicin, pazopanib	2	0.4
Etoposide, ifosfamide, methotrexate	1	0.2
Gemcitabine, ifosfamide	1	0.2
Gemcitabine, sunitinib	1	0.2
Ifosfamide, paclitaxel	1	0.2
Methotrexate	1	0.2
Pazopanib, sorafenib	1	0.2
Bevacizumab, irinotecan	1	0.2
Bevacizumab, irinotecan, temozolomide, vincristine	1	0.2
Bevacizumab, paclitaxel	1	0.2
Bevacizumab, docetaxel	1	0.2
Bevacizumab, doxorubicin	1	0.2
Temozolomide, topotecan, vincristine	1	0.2
Carboplatin, etoposide, irinotecan	1	0.2
Carboplatin, gemcitabine, paclitaxel	1	0.2
Carboplatin, methotrexate, paclitaxel	1	0.2
Carboplatin, epirubicin, paclitaxel	1	0.2
Cyclophosphamide, dacarbazine, doxorubicin, vincristine	1	0.2
Cyclophosphamide, doxorubicin	1	0.2
Cyclophosphamide, doxorubicin, etoposide, vincristine	1	0.2
Cyclophosphamide, doxorubicin, paclitaxel	1	0.2
Dacarbazine, gemcitabine	1	0.2
Dacarbazine, pazopanib	1	0.2
Dacarbazine, doxorubicin, gemcitabine	1	0.2
Dacarbazine, doxorubicin, ifosfamide	1	0.2
Dacarbazine, doxorubicin, methotrexate	1	0.2
Docetaxel, paclitaxel	1	0.2
Docetaxel, doxorubicin, gemcitabine, ifosfamide	1	0.2
Doxorubicin, gemcitabine, vinorelbine	1	0.2
Doxorubicin, ifosfamide, pazopanib	1	0.2
Doxorubicin, ifosfamide, vincristine	1	0.2
LOT 2 regimen		
Methotrexate	7	3.1
Paclitaxel	7	3.1
Carboplatin, paclitaxel	7	3.1
Doxorubicin, ifosfamide	5	2.2
Gemcitabine, paclitaxel	4	1.7
Ifosfamide	4	1.7
Sorafenib	4	1.7
Vinorelbine	4	1.7
Dacarbazine, doxorubicin	4	1.7
Docetaxel	4	1.7
Bevacizumab	3	1.3
Carboplatin	3	1.3
Dacarbazine	3	1.3
Etoposide	2	0.9
Bevacizumab, paclitaxel	2	0.9
Bevacizumab, temozolomide	2	0.9
Bevacizumab, docetaxel, gemcitabine	2	0.9
Carboplatin, gemcitabine	2	0.9
Doxorubicin, gemcitabine	2	0.9
Eribulin	2	0.9
Etoposide, ifosfamide	1	0.4
Etoposide, ifosfamide, irinotecan, vincristine	1	0.4
Gemcitabine, irinotecan	1	0.4
Gemcitabine, pazopanib	1	0.4
Gemcitabine, vinorelbine	1	0.4
Irinotecan, temozolomide	1	0.4
Irinotecan, temozolomide, vincristine	1	0.4
Paclitaxel, pazopanib	1	0.4
Pazopanib, sunitinib	1	0.4
Sunitinib	1	0.4
Bevacizumab, dacarbazine	1	0.4
Bevacizumab, doxorubicin	1	0.4
Topotecan	1	0.4
Carboplatin, methotrexate, paclitaxel	1	0.4
Cyclophosphamide, etoposide	1	0.4
Cyclophosphamide, methotrexate	1	0.4
Cyclophosphamide, temozolomide, topotecan	1	0.4
Cyclophosphamide, topotecan	1	0.4
Cyclophosphamide, vincristine	1	0.4
Cyclophosphamide, dacarbazine, doxorubicin	1	0.4
Cyclophosphamide, dacarbazine, doxorubicin, vincristine	1	0.4
Cyclophosphamide, docetaxel	1	0.4
Cyclophosphamide, doxorubicin, etoposide, ifosfamide	1	0.4
Cyclophosphamide, doxorubicin, etoposide, ifosfamide, vincristine	1	0.4
Cyclophosphamide, doxorubicin, vincristine	1	0.4
Dacarbazine, gemcitabine	1	0.4
Dacarbazine, ifosfamide	1	0.4
Dacarbazine, doxorubicin, ifosfamide	1	0.4
Docetaxel, gemcitabine, paclitaxel	1	0.4
Docetaxel, gemcitabine, pazopanib	1	0.4
Docetaxel, doxorubicin, gemcitabine	1	0.4
Doxorubicin, gemcitabine, vinorelbine	1	0.4
Doxorubicin, paclitaxel	1	0.4
Doxorubicin, pazopanib	1	0.4
LOT 3 regimen		
Vinorelbine	4	4.5
Gemcitabine	3	3.4
Paclitaxel	2	2.3
Cyclophosphamide, dactinomycin, vincristine	2	2.3
Cyclophosphamide, doxorubicin, vincristine	2	2.3
Docetaxel	2	2.3
Etoposide, ifosfamide	1	1.1
Etoposide, ifosfamide, irinotecan	1	1.1
Gemcitabine, irinotecan	1	1.1
Gemcitabine, methotrexate	1	1.1
Ifosfamide	1	1.1
Irinotecan, temozolomide	1	1.1
Irinotecan, vincristine	1	1.1
Sorafenib	1	1.1
Bevacizumab, gemcitabine, paclitaxel	1	1.1
Bevacizumab, cyclophosphamide, sorafenib	1	1.1
Carboplatin	1	1.1
Topotecan	1	1.1
Cyclophosphamide, topotecan	1	1.1
Cyclophosphamide, doxorubicin	1	1.1
Cyclophosphamide, doxorubicin, irinotecan, vincristine	1	1.1
Dacarbazine	1	1.1
Dacarbazine, pazopanib	1	1.1
Dacarbazine, vinorelbine	1	1.1
Dacarbazine, doxorubicin	1	1.1

## Abbreviations

mSTS: metastatic soft tissue sarcoma
NCCN: National Comprehensive Cancer Network
LOT: line of therapy
STS: soft tissue sarcoma
FDA: Food and Drug Administration
SABINE: Sarcoma Treatment and Burden of Illness in North America and Europe
ORD: Optum Research Database
SSA: Social Security Administration
GIST: gastrointestinal stromal tumors
ICD-9-CM: International Classification of Diseases, 9th revision, Clinical Modification
CPT: Current Procedural Terminology
HCPCS: Healthcare Common Procedure Coding System
MS-DRG: Medicare Diagnosis-Related Group
CI: confidence interval
